# Identification of two novel activities of the Wnt signaling regulator Dickkopf 3 and characterization of its expression in the mouse retina

**DOI:** 10.1186/1471-2121-8-52

**Published:** 2007-12-19

**Authors:** Rei EI Nakamura, Dale D Hunter, Hyun Yi, William J Brunken, Abigail S Hackam

**Affiliations:** 1 Bascom Palmer Eye Institute, University of Miami Miller School of Medicine, Miami, FL, USA; 2 Neuroscience Graduate Program, University of Miami Miller School of Medicine, Miami, FL, USA; 3 Department of Neuroscience, Tufts University School of Medicine, Boston, MA, USA; 4 Department of Anatomy and Cell Biology, State University of New York, Downstate Medical Center, Brooklyn, NY, USA

## Abstract

**Background:**

The Wnt signaling pathway is a cellular communication pathway that plays critical roles in development and disease. A major class of Wnt signaling regulators is the Dickkopf (Dkk) family of secreted glycoproteins. Although the biological properties of Dickkopf 1 (Dkk1) and Dickkopf 2 (Dkk2) are well characterized, little is known about the function of the related Dickkopf 3 (Dkk3) protein in vivo or in cell lines. We recently demonstrated that Dkk3 transcripts are upregulated during photoreceptor death in a mouse model of retinal degeneration. In this study, we characterized the activity of Dkk3 in Wnt signaling and cell death.

**Results:**

Dkk3 was localized to Müller glia and retinal ganglion cells in developing and adult mouse retina. Western blotting confirmed that Dkk3 is secreted from Müller glia cells in culture. We demonstrated that Dkk3 potentiated Wnt signaling in Müller glia and HEK293 cells but not in COS7 cells, indicating that it is a cell-type specific regulator of Wnt signaling. This unique Dkk3 activity was blocked by co-expression of Dkk1. Additionally, Dkk3 displayed pro-survival properties by decreasing caspase activation and increasing viability in HEK293 cells exposed to staurosporine and H_2_O_2_. In contrast, Dkk3 did not protect COS7 cells from apoptosis.

**Conclusion:**

These data demonstrate that Dkk3 is a positive regulator of Wnt signaling, in contrast to its family member Dkk1. Furthermore, Dkk3 protects against apoptosis by reducing caspase activity, suggesting that Dkk3 may play a cytoprotective role in the retina.

## Background

Wnt ligands are secreted glycoproteins that control a wide range of processes in the developing embryo and in adult tissues. Aberrant Wnt signaling is increasingly being implicated in numerous diseases, including malignancies, Alzheimers disease, retinal degenerations and abnormal development of the eye, limbs and skeleton [1-3]. Characterizing proteins that regulate the Wnt pathway have revealed important insights into Wnt-dependent processes and potential directions towards novel therapies [4].

The best understood of the major Wnt pathways is the canonical/β-catenin pathway. In the absence of Wnt ligands, β-catenin levels are suppressed by the APC-axin-GSK3β protein complex via phosphorylation and subsequent degradation by the proteosome [5]. β-catenin is a transcriptional cofactor and is also an essential component of cell-cell adhesion complexes. Wnt ligands bind to the cell surface receptors Frizzled and LDL receptor related proteins 5 and 6 (LRP5/6), leading to Disheveled activation and ultimately reducing β-catenin degradation. Stabilized β-catenin is transported into the nucleus where it binds to Tcf/Lef type transcription factors and initiates transcription of Wnt target genes.

The Dickkopf (Dkk) family of proteins, Dkk1, 2, 3 and 4 and Soggy, are secreted regulators of Wnt signaling [6-8]. The five Dkk proteins share 37–50% protein identity and contain two conserved cysteine-rich regions separated by a variable linker region [8]. Dkk1, Dkk2 and Dkk4 inhibit Wnt signaling by binding to LRP5/6 and the transmembrane protein Kremen which results in LRP5/6 internalization and prevents Wnt and Frizzled from forming an active complex with LRP5/6 [9,10]. Dkk2 can also activate the Wnt pathway in certain situations, depending on the cell type, the presence of Wnt ligands and levels of LRP5/6 [11-13].

Unlike its related family members, characterizing Dkk3 activity has been elusive. Dkk3 did not regulate Wnt signaling in various activity assays, including Wnt-dependent secondary axis induction in *Xenopus *embryos and Wnt1/Fz8 signaling in cultured cells [8,11,12]. Dkk3 also did not physicallyinteract with LRP5/6 or Kremen [9,14]. However, Caricasole et al demonstrated that Dkk3 was a weak inhibitor of Wnt7A signaling in PC12 cells although co-expression of LRP5 or LRP6 was required to uncover this activity [15]. Dkk3 displayed Wnt inhibitor activity in the osteocarcinoma Saos-2 cell line, measured by decreased cytoplasmic levels of β-catenin [16], but did not inhibit Wnt reporter Tcf/Lef luciferase activity assays in a prostate cancer cell line [17]. Therefore, the relationship between Dkk3 and Wnt signaling is unclear despite its sequence similarity to the other Dkk genes.

Dkk3 is expressed during embryonic development in many organs, including neural epithelium, limb bud, bone and heart, particularly in regions of epithelial-mesenchyme transformation [18]. Dkk3 is also widely expressed in adult tissues, with the highest levels found in the heart and brain [8]. *Dkk3*-deficient mice develop normally, are fertile and have a mild phenotype that includes hyperactivity, increased immunological and hematological markers and a slight decrease in lung ventilation [19]. The absence of severe phenotypes in Dkk3 knock-out mice may be due to compensation from the Dkk3 homolog *soggy *[19]. Alternatively, physiological stress or injury may be required for the appearance of a Dkk3-dependent phenotype.

In this study, we investigated the activity of Dkk3 in Wnt signaling and cell death. We demonstrated two novel functions for Dkk3. First, Dkk3 is a cell-specific positive regulator of the canonical Wnt signaling pathway in primary cell culture and cell lines. Second, Dkk3 protected transfected cells from apoptotic stress. We also characterized the distribution of Dkk3 in the retina and found that Dkk3 is expressed highly in Müller glia and ganglion cells during retinal development and in adult retina. Müller glia are the principle supportive glia in the retina and are believed to protect photoreceptors during retinal injury by secreting growth factors [20-23]. We previously demonstrated that Dkk3 transcripts were increased in a mouse model of retinal degeneration, particularly during cone photoreceptor death [24]. Furthermore, Wnt signaling is upregulated during retinal degeneration in Müller glia and Wnt activators protect primary photoreceptor cultures from apoptosis [25]. Together, these results identify for the first time that Dkk3 is a secreted pro-survival signaling protein, and suggest that Dkk3 may play a role in Müller glia activity in the retina.

## Results

### Dkk3 is expressed and secreted from retinal Müller glia

**Figure 1:**
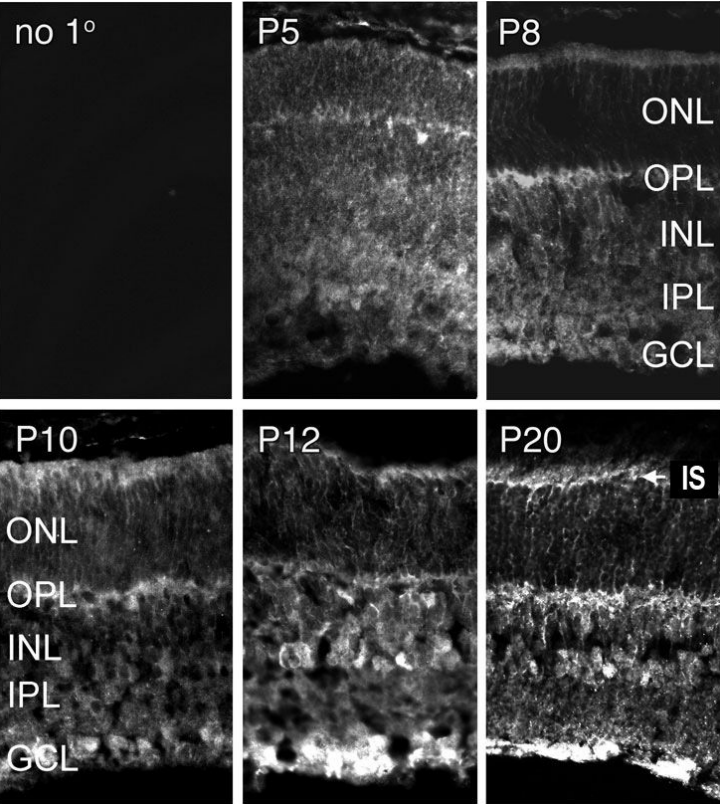
**Dkk3 protein is expressed in the retina during Müller glial cell differentiation**. Dkk3 immunoreactivity is present diffusely in the developing retina at P5 and at P8 it is localized to the outer plexiform layer (OPL), the site of synaptic processes between photoreceptors and interneurons, and the ganglion cell layer (GCL), where it remains at P10 and P12. During the later stages of retinal differentiation Dkk3 becomes predominantly localized to the inner nuclear layer (INL) and at the inner segment region (IS, arrow), consistent with localization to Müller glia cell bodies and end-feet. Expression in the OPL and GCL remains high at these ages. By P20, Dkk3 appears localized along radial fibers, representing Müller glia processes, extending across the retina. These fibers are particularly visible in the outer nuclear layer (ONL). IPL, inner plexiform layer.

Cellular differentiation in the mouse retina into the major neuronal and glial cell types occurs at stereotypic time-points during the neo-natal period. We used immunohistochemistry to characterize the distribution of Dkk3 during post-natal retinal development, especially during the time of Müller glia differentiation. Dkk3 immunoreactivity is present diffusely at post-natal day (P) 5 and becomes concentrated in the developing synaptic layers in the outer plexiform layer (OPL) and ganglion cell layer (GCL) by P8, and then remains in these locations through the beginning of Müller glia differentiation (P10-12) (Figure 1). As Müller glia cells continue to differentiate during P12 to P20, Dkk3 becomes more concentrated in the inner nuclear layer (INL), where nuclei of the interneurons horizontal cells, bipolar cells and amacrine cells are located, and in the narrow margin composed of the photoreceptor inner segments and the external limiting membrane. Localization to this inner segment region (IS) suggests that Dkk3 is expressed in or secreted from Müller glia end-feet. By P20, Dkk3 appears associated with radial fibers extending across the retina, which correspond to Müller glia processes.

**Figure 2:**
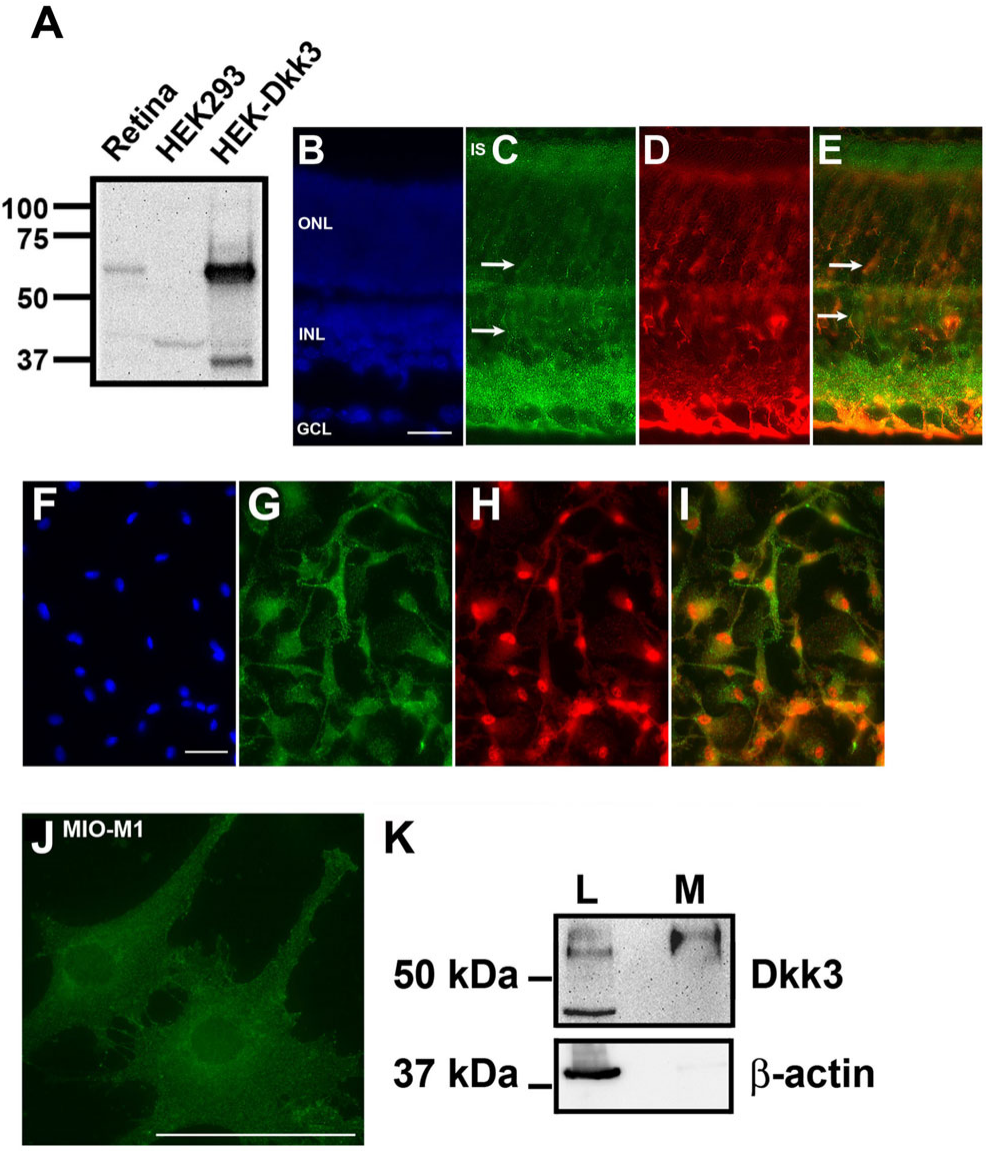
**Dkk3 is expressed in adult mouse retina, primary Müller glia culture and a human Müller glial cell line**. (A) Western blot on lysates from retina, HEK293 cells and a stable cell line expressing Dkk3 (HEK-Dkk3). A single band at 58 kDa, representing post-translationally modified Dkk3, is observed in the retina and in the Dkk3 stable cell line. Smaller bands likely represent degradation products or partially glycosylated Dkk3. (B-E) Dkk3 (green) is located in the photoreceptor inner segment region (IS), inner nuclear layer (INL) and ganglion cell layer (GCL) of adult wild type mice. There is also strong staining in the inner plexiform layer between the INL and GCL. The Müller glia marker glutamine synthetase (GS) is red and DAPI-stained nuclei are blue. The outer nuclear layer (ONL) contains the photoreceptor nuclei. Dkk3 colocalizes with GS-positive Müller glia radial fibers and cell bodies (Figure 2E,C arrows). (F-I) Confirmation of Dkk3 localization in Müller glia. Rat primary Müller glia were isolated from the retina and cultured. Dkk3 (green) is found in the same cells as the Müller glia marker glutamine synthetase (red). DAPI-stained nuclei are blue and the merged image is shown. (G) Dkk3 (green) is expressed in the perinuclear region and along processes in the human Müller glial cell line MIO-M1. Scale bar in B and F is 20 μm and in J is 100 μm. (H) Dkk3 is secreted from MIO-M1 cells. Precipitated proteins from the culture media were separated on a Western blot. The 58 kDa glycosylated Dkk3 is present in both cell lysate (L) and media (M) whereas the 38 kDa non-glycosylated form is not secreted and is only in the cell lysate fraction. β-actin was used as a control.

Dkk3 remained localized in the inner nuclear layer and the ganglion cell layer in adult mouse retina (Figure 2B–E), consistent with our previous study localizing Dkk3 transcript distribution [24]. Dkk3 staining in the adult also remained prominent in the synaptic plexiform layers between the ONL and INL, and the INL and GCL, and the photoreceptor inner segment (IS) region. Double immunostaining using antibodies against Dkk3 and the Müller glia marker glutamine synthetase (GS) indicate that Dkk3 colocalizes with GS-positive Müller glia radial fibers and cell bodies (Figure 2C,E, arrows). The immunostaining in Figures 1 and 2 used different anti-Dkk3 antibodies (see Methods) and the equivalent staining patterns confirm the specificity of Dkk3 detection. The specificity of the antibody was confirmed by Western blotting, which detected a single band at the predicted size (Figure 2A,K).

Müller glia span the entire thickness of the tissue, ensheathing and interacting with all retinal neurons. Müller glia are vital to the normal retinal function by regulating ion concentrations, signaling molecules and neurotransmitters, mediating water transport, and removing glutamate and scavenging free radicals. During retinal degeneration Müller glia are believed to secrete pro-survival growth factors that protect remaining photoreceptors from injury. To confirm Dkk3 expression in glia we cultured Müller glia from rat and mouse retinas. One hundred percent of the Müller glia showed colocalization of Dkk3 with the Müller glia marker protein glutamine synthetase (Figure 2F–I). Dkk3 was distributed along processes and also in a punctate peri-nuclear pattern, consistent with the endoplasmic reticulum/Golgi complex. This peri-nuclear localization is evident in a higher magnification image of the human Müller glia cell line MIO-M1 [26] (Figure 2J). Western blotting confirmed that Dkk3 is secreted (M, media) from MIO-M1 cultures (Figure 2K).

### Dkk3 regulates Wnt signaling

Wnt signaling was measured using a transcriptional reporter assay with the TOP-FLASH luciferase plasmid. MIO-M1 Müller glia cells were cotransfected with TOP-FLASH and LacZ plasmids along with Dkk1, Dkk3 or a control gene (GFP). At 48 hrs post-transfection, the transfected cells were incubated with Wnt3a-containing conditioned media or control conditioned media that lacks the Wnt3a ligand.

**Figure 3:**
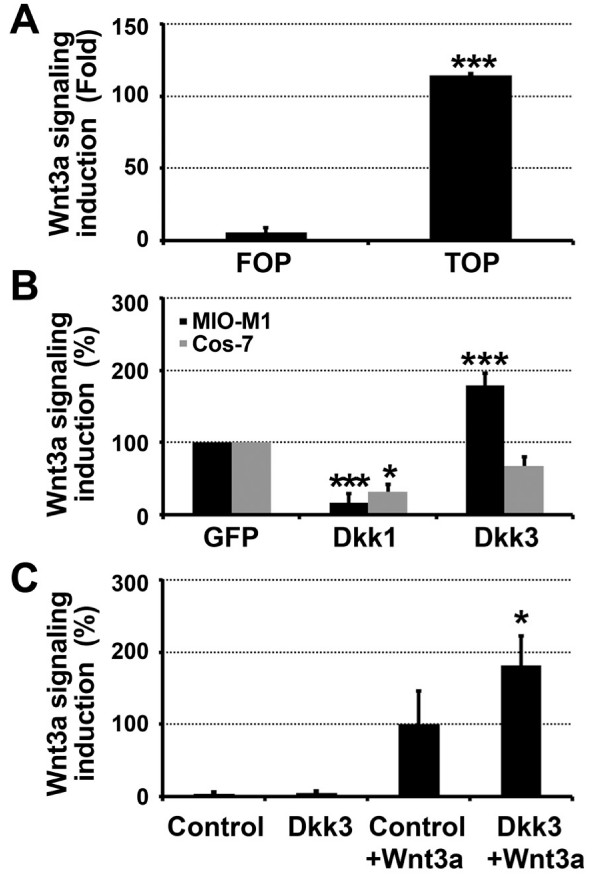
**Dkk3 is a positive regulator of Wnt signaling in Müller glia and a negative regulator in COS7 cells**. (A) Wnt signaling was induced by Wnt3a conditioned media in the MIO-M1 human Müller glia cell line, measured using the TOP-FLASH Wnt luciferase reporter assay. The mutated control reporter FOP-FLASH did not show a response to Wnt3a. Induction is expressed as fold change of conditioned media containing Wnt3a to conditioned media not containing Wnt3a. (B) Luciferase reporter assays in cells transfected with Dkk1, Dkk3 and a control gene (GFP). Dkk3 increased Wnt3a-induced Wnt signaling whereas Dkk1 decreased Wnt signaling in the MIO-M1 human Müller glia cell line (black bars). Wnt signaling was measured using the TOP-FLASH luciferase assay. Wnt signaling is shown relative to GFP-expressing cells. In the COS7 cell line (grey bars), Dkk1 reduced Wnt signaling and Dkk3 had a small but not significant inhibitory effect. (C) Primary Müller glia cultured from the mouse retina that were treated with conditioned media with or without Dkk3 had baseline luciferase levels. Incubation with Wnt3a showed a significant induction of Wnt signaling that was greater when Dkk3 was present (Dkk3+Wnt3a) than when Wnt3a was added alone without Dkk3 (Control+Wnt3a). * = p < 0.05, *** = p < 0.001, relative to GFP-expressing cells. Mean ± standard deviation is shown.

Wnt signaling was induced by Wnt3a conditioned media in the MIO-M1 human Müller glia cell line (Figure 3A), indicating that these cells are Wnt-responsive, as we recently demonstrated [25]. The mutated control reporter FOP-FLASH did not show a response to Wnt3a, as expected (Figure 3A). Dkk1 reduced Wnt3a-induced signaling in MIO-M1 cells by approximately five-fold (Figure 3B) compared with GFP expression. In contrast, Dkk3 induced approximately two-fold higher Wnt signaling compared with GFP (Figure 3B). Interestingly, Dkk3 potentiating activity was cell-type specific. In contrast to the MIO-M1 cells, Dkk3 had a small inhibitory, although not significant, effect on Wnt3a-induced signaling in the COS7 cell line and reduced luciferase activity by 33%compared with control tr ansfections (Figure 3B). Dkk1 decreased luciferase activity by 67% in COS7 cells (Figure 3B). This result suggests that COS7 cells do not have the molecular mechanisms to convey Dkk3 potentiating activity or that the inhibitor activity of Dkk3 is greater than, and over-rides, its potentiating activity.

Dkk3 increased Wnt3a-induced signaling in primary Müller glia isolated from mouse retina. In these experiments we tested the activity of Dkk3 when it was added to the glia cells as conditioned media. HEK293 cells stably expressing Dkk3 were created and used as a source of Dkk3 [2] (see Methods). Dkk3 treatment in the absence of Wnt3a produced baseline levels of Wnt signaling and was equivalent to control media not containing Dkk3 (Figure 3C). Dkk3 by itself had baseline levels in all the cell types tested, indicating that Dkk3 potentiates the Wnt pathway when it is stimulated by Wnt3a but cannot initiate signaling on its own.

Incubating primary Müller glia in Dkk3-containing conditioned media increased Wnt signaling by 1.8-fold (Dkk3+Wnt3a compared with Wnt3a treatment alone (control+Wnt3a)). (Figure 3C). Although co-stimulation with a Wnt ligand was required to demonstrate Dkk3 activity, over-expression of Wnt receptors was not necessary, in contrast to Dkk3 activity in PC12 cells [15].

To begin to explore the mechanism of Wnt regulation by Dkk3, we tested whether Dkk1 blocks Dkk3 activity. For this experiment we used a Dkk3 stable cell line made in HEK293 cells to ensure that the majority of cells coexpressed Dkk3 and Dkk1. We previously demonstrated that Dkk3 potentiated Wnt signaling in this cell type[2].

**Figure 4:**
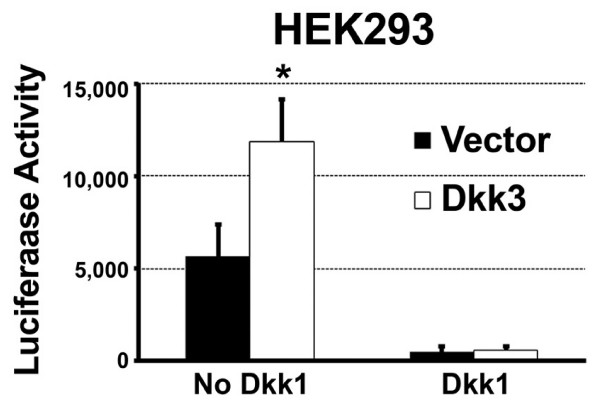
**Dkk1 blocks Dkk3 activity**. Dkk3 (white bars) and control (black bars) stable cell lines (HEK293 cells) were transfected with the control vector ("no Dkk1") and TOP-FLASH and LacZ plasmids then treated with Wnt3a. The Dkk3 cell line had more Wnt signaling than the vector control line (Luciferase Activity units shown). Transfection of Dkk1 completely abolished Wnt signaling in both the Dkk3 stable cell line and the control cell line. This result suggests that Dkk3 acts in a Dkk1-dependent pathway. * = p < 0.05. Mean ± standard deviation is shown.

Wnt3a ligand induced significantly more Wnt signaling in the cells expressing Dkk3 than the control cell line made with the empty vector (Figure 4, compare white and black bars in cells without Dkk1). If Dkk3 acts independently from Dkk1 then we expect that Dkk1 would not fully inhibit Wnt signaling in Dkk3-expressing cells compared with the vector cell line, and that the combination of Dkk3 + Dkk1 + Wnt3a would be less inhibited and have more Wnt signaling than Dkk1 + Wnt3a in the vector cell line. As shown in Figure 4, Dkk1 inhibited Wnt3a-induced signaling to an equivalent extent in the Dkk3 and vector stable cell lines. This result suggests that Dkk3-mediated potentiation of Wnt3a signaling is dependent on, or occurs upstream from, a Dkk1-controlled pathway or receptor.

Finally, it is unlikely that the transfection method influenced the effect of Dkk3 because there is no correlation between Dkk3 activity and lipofectamine or electroporation. For example, Dkk3 had opposite effects in HEK293 and COS7 cells despite both being transfected with lipofectamine, whereas Dkk3 activity was similar in MIO-M1 cells as HEK293 despite the former being transfected using electroporation.

### Dkk3 regulates cell death

**Figure 5:**
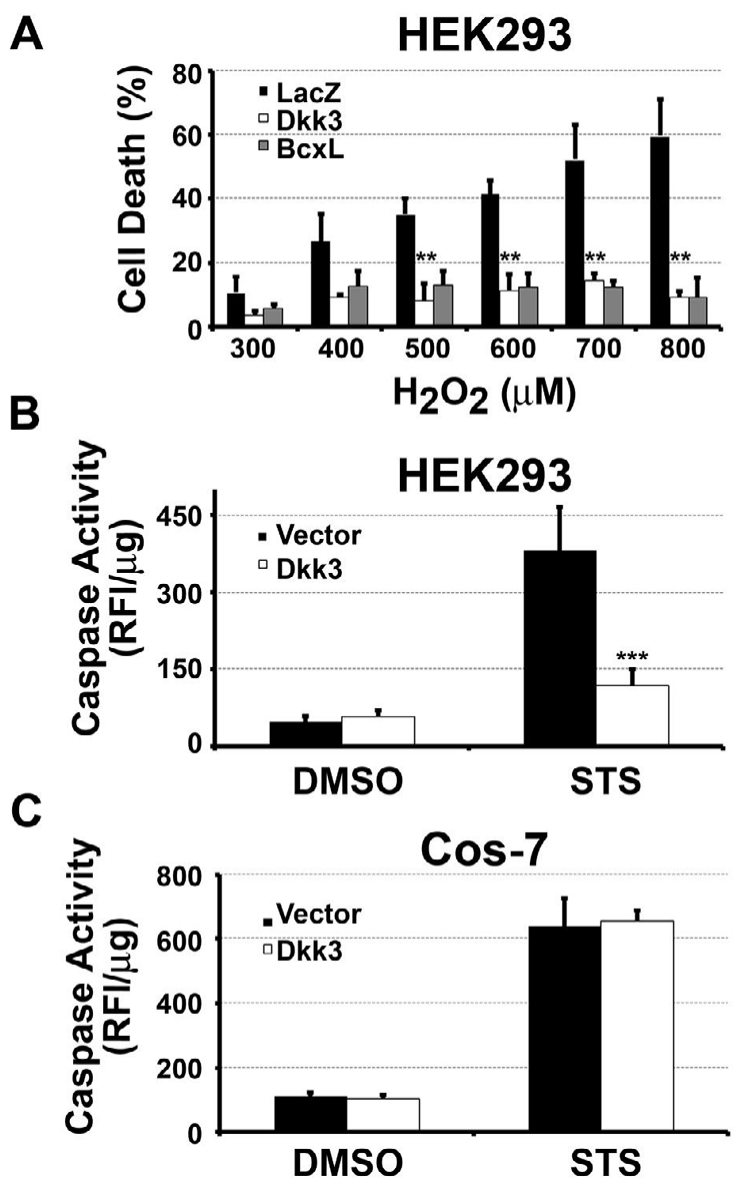
**Dkk3 regulates cell death**. (A) HEK293 cells were transiently transfected with LacZ, Dkk3, or BclxL and exposed to H_2_O_2 _for 24 hr. Viability was measured by XTT assay and was normalized to 0 mM H_2_O_2_. Dkk3 expression significantly increased viability in the presence of H_2_O_2 _compared with LacZ control. There was no difference between Dkk3 and Bcl_XL_. ** = p < 0.001, comparison between Dkk3 and LacZ. The comparison between Bcl_XL _and LacZ was also significant at p < 0.001 at each concentration of H_2_O_2_. (B) Dkk3 significantly protected HEK293 cells against apoptosis induced by staurosporine. HEK293 cells expressing Dkk3 or Vector control were exposed to 1 μM staurosporine (STS) or the DMSO vehicle control for 16 hrs. Caspase activity was measured and relative fluorescent intensity (RFI) units were normalized to micrograms of protein. *** = p < 0.001, comparison between Dkk3 cells and Vector cells treated with STS. (C) Dkk3 did not protect COS7 cells exposed to staurosporine. Caspase activity (RFI/microgram of protein) was compared with cells transfected with the pcDNA3 empty vector. Mean ± standard deviation is shown.

We next investigated whether Dkk3 plays a role in cell survival by testing the effect of Dkk3 expression on cell death induced by H_2_O_2 _and staurosporine. The HEK293 cell line was used because it has Dkk3-responsive Wnt signaling (Figure 4 and [2]), indicating the potential presence of Dkk3 receptors and signaling proteins. HEK293 cells were transiently transfected with Dkk3 or the control genes LacZ or Bcl_XL _and were exposed to 0.3 to 0.8 mM H_2_O_2 _for 24 hr. Dkk3 expression significantly increased viability in cells exposed to H_2_O_2_, compared with the LacZ transfection control (Figure 5A). Indeed, the pro-survival effect of Dkk3 was equivalent to the anti-apoptotic gene Bcl_XL _used as the positive control. The baseline viability of HEK293 cells transfected with Dkk3 and Vector were equivalent in the absence of apoptotic inducers (data not shown).

Dkk3 transfection also decreased apoptosis induced by staurosporine, a well-characterized pro-apoptotic protein kinase inhibitor. Dkk3 expression reduced caspase activation by 4-fold in HEK293 cells, compared with the vector control (Figure 5B). Protection from apoptosis was also observed in three independent clones of HEK293 cell lines stably expressing Dkk3 (data not shown). However, Dkk3 did not reduce caspase activity in COS7 cells (Figure 5C). Therefore, Dkk3 demonstrates cell-specific protection, similar to its cell-specific potentiation of Wnt signaling.

## Discussion and Conclusion

The function of Dkk3 and its role in Wnt signaling had been unclear despite it sharing approximately 40% protein identity to the well-characterized Dkk1 and Dkk2 Wnt inhibitors. In the present study, we found that Dkk3 is a cell-type specific regulator of Wnt signaling. Dkk3 increased Wnt activation in primary Müller glia cultures, a Müller glia cell line and in HEK293 cells, but not in COS7 cells. Dkk3 activity was not dependent on the method of delivery because transient and stable transfections of Dkk3 plasmids and treatment with Dkk3 conditioned media demonstrated equivalent results. Although the effect of Dkk3 on Wnt signaling may be indirect, to our knowledge this is the first demonstration that Dkk3 acts as a cell-specific positive regulator of the Wnt pathway.

Dkk3 was originally proposed to be a divergent, non-Wnt regulating Dkk family member [8] although later studies indicated that Dkk3 inhibits Wnt signaling in PC12 and osteocarcinoma Saos-2 cells [15,16]. Discrepant effects on the Wnt pathway have been observed for other Wnt regulator genes. For example, Dkk2 activates Wnt signaling when coexpressed with certain Frizzled and LRP6 receptors but inhibits Wnt signaling in other situations [12,13]. Wnt1 also activates canonical Wnt signaling in some cell lines but inhibits Wnt signaling in others [27]. Furthermore, the non-canonical Wnt/Ca^2+ ^pathway activator Wnt5a activates canonical Wnt signaling in HEK293 cells when the co-receptors Fz4 and LRP5 are over-expressed, but inhibits canonical signaling in the absence of these receptors [28]. Therefore, Wnt signaling activation in vitro, and potentially also in vivo, is not a sole property of the ligand or regulator but appears to be related to other factors, including receptor availability [28].

Receptor context influences the regulation of canonical Wnt signaling by Dkk2 [11] and Wnt5a [28], suggesting that specific receptor components in HEK293 and Müller glia, such as a putative Dkk3 receptor, may mediate the Wnt-inducing activity of Dkk3. Absence or lower expression of these components may cause its inhibitory activity in COS7 (Figure 4) and PC12 cells [15] and its inactivity in other assays. Although Dkk3 activity was blocked by Dkk1 coexpression in HEK293 cells, the mechanism of Wnt modulation by Dkk3 may differ from Dkk1 and Dkk2 because Dkk3 does not bind to Kremen or LRP proteins [9,14]. Future studies will investigate the factors that are required for Dkk3 activity.

We demonstrated that Dkk3 has pro-survival properties in HEK293 cells but not in COS7 cells. This cellular difference between Dkk3 protection also suggests that COS7 cells lack the appropriate Dkk3 receptors, although this cell type does express endogenous Dkk3 (data not shown). Although the Wnt pathway itself is protective in most tissues [29,30], Dkk3-mediated cellular protection is unlikely to be dependent on Wnt signaling because Wnt3a was not added in the apoptosis assays. However, we cannot exclude the possibility that low levels of Wnt activators are present in the culture serum.

Several reports demonstrated that Dkk3 regulates cell death in cancer cells, protecting osteocarcinoma cells against serum starvation and chemotherapy agents [16] but inducing apoptosis in liver cancer cell lines [31]. In our study, Dkk3 expression alone did not increase basal viability, suggesting that it plays a reactive role after apoptosis has begun. Interestingly, *dkk3*-deficient mice did not display any phenotypes that would indicate defects in cell survival or altered Wnt signaling [19]. Furthermore, these mice had normal retinal development. If Dkk3 is protective only in response to retinal injury then tissue damage may be required to reveal a phenotype. Alternatively, Dkk3-mediated protection may be limited to specific cell types in culture.

Müller glia secrete pro-survival growth factors during retinal degeneration that are believed to rescue remaining photoreceptors in a toxic environment [22,32-34]. We recently demonstrated that Wnt signaling is upregulated during retinal degeneration in Müller glia and Wnt activators protect primary photoreceptor cultures from apoptosis [25]. The localization of Dkk3 to Müller glia and its increased expression during retinal degeneration, combined with the functional properties identified above, raise the intriguing possibility that Dkk3 may be a component of Müller glia-mediated retinal protection. Such protection could occur directly by acting on vulnerable photoreceptors, or indirectly by activating Wnt signaling. The glia may secrete Dkk3 in vivo which could then increase Wnt signaling in fellow glia or in neighboring cell types. Although the cell lines are not an appropriate model for photoreceptors, we have achieved our goal of defining roles of Dkk3 in cultured cells as a first approach towards determining the potential activity of Dkk3 in the retina. Manipulating Dkk3 expression in normal and degenerating retina will provide insight the role of Dkk3 in the retina.

## Methods

### Reagents

Dkk1 and Dkk3 expression vectors were generously provided by Dr. Niehrs (Heidelberg, Germany) [6]. The HEK293, COS7, MIO-M1 cell lines and the primary Müller glia cultures were maintained in DMEM growth medium supplemented with 10% fetal bovine serum, 100 units/ml of penicillin and 100 μg/ml of streptomycin at 37°C in 5 % CO_2_. The Dkk3 stable cell line was established by transfecting HEK293 cells with Dkk3 in the pcDNA3 vector and selecting for clones using geneticin [2]. The control "Vector" line was made in parallel by transfecting with the empty vector. Transfections used Lipofectamine (Invitrogen), electroporation or buffered calcium phosphate, according to standard procedures.

Canonical Wnt signaling was induced by incubation with conditioned media (CM) prepared from mouse L-cells stably expressing Wnt3a (ATCC). The CM was filtered and mixed 1:1 with normal media for use. Dkk3-containing conditioned media was prepared from HEK293 cells stably expressing Dkk3. The control conditioned media for Wnt3a was from parental L-cells and for Dkk3 it was from the HEK293 "Vector" cell line. The following primary antibodies were used: rabbit anti-glutamate synthetase (Sigma, 1:500), Dkk3 polyclonal antibody (Santa Cruz, 1:100) and Dkk3 monoclonal antibody (R&D, 1:500).

### Wnt activity assays

Cells were co-transfected with the Dkk plasmid, the TOP-FLASH luciferase reporter plasmid (a generous gift from Dr. R. Moon, University of Washington) and a LacZ-containing plasmid. The endogenous β-catenin/Tcf transcriptional complex binds to Tcf sequences upstream of the enzyme luciferase in the TOP-FLASH plasmid vector, driving luciferase expression upon Wnt pathway activation. The mutated FOP-FLASH plasmid was used as a control and did not have luciferase activity in transfected cells treated with Wnt3a (Figure 2A). Forty-eight hours after transfection the cells were incubated with Wnt3a conditioned media or control conditioned media for 16–24 hours and lysates were collected in Reporter lysis buffer (Promega). Luciferase activity was measured in a Lumistar Galaxy luminometer (BMG Labtech) and normalized to β-galactosidase activity [2]. The assays were performed in duplicate in at least five independent experiments. Values were normalized to treatment with media not containing Wnt3a.

### Viability Assays

Caspase assays used the Ac-DEVD-AMC substrate (Alexis Biochemicals) and were performed according to the manufacturer's instructions. Briefly, approximately 5 × 10^5 ^cells were transfected in a 6-well plate and transfected. At 24 hours post-transfection apoptosis was induced by 1 μM staurosporine. The cells were lysed in RIPA buffer (50 mM Tris-HCl, pH 7.4, 1 % NP40, 0.25 % sodium deoxycholate, 150 mM NaCl, 1 mM EDTA, 1 mM sodium orthovanadate). 50 μL of DEVD-AMC reaction buffer mixture containing 40 mM HEPES pH 7.5, 20% glycerol, 4 mM DTT and 0.2 mM of the fluorogenic caspase 3 substrate Ac-DEVD-AMC was added to cellular lysates for 4 hour at 37°C, and fluorescence was then measured at 380 nm excitation and 450 nm emission. Fluorescent intensity values were normalized to protein concentration, measured using the Lowry assay (BioRad).

Cell viability was measured using the XTT assay [35]. Cells were seeded into 96-well plates and triplicate wells were transfected with Dkk3, Bcl_XL _or LacZ. H_2_O_2 _was added for 24 hrs and then 10 μl WST-1 reagent (Roche) was added for 1 hour and the release of formazan from mitochondria was quantified at 450 nm using an ELISA plate reader. Each experiment was performed at least three times on different days with three to five replicates within an experiment.

### Primary Müller glia culture

All procedures involving animals were performed in accordance with theARVO Statement for the Use of Animals in Ophthalmic and Vision Research and were approved by the Animal Care and Use Committee at the University of Miami Miller School of Medicine. Mouse and rat Müller glia cultures were prepared from retinas from animals from P4-P8. Retinas were dissociated with papain and seeded onto cell culture plates. After 16 hr, nonattached cells (mostly neuronal) were removed by gentle agitation [25,36]. Immunohistochemistry using antibodies against retinal cell-specific markers was used to assess the purity of the culture, as described in [25]. One hundred percent of the cells in the culture were identified as Müller glia based on morphology and immunostaining for the Müller glia marker glutamine synthetase (GS) and the lack of immunodetection for other cell-specific marker proteins. After one week in culture the cells were trypsinized and equal numbers of cells were plated for transfection using electroporation.

### Immunohistochemistry

Eyes were enucleated from C57Bl/6 mice and immediately fixed in 4% paraformaldehyde, incubated in increasing sucrose concentrations (5–20%), embedded in OCT and flash-frozen. Sections (10 μm) were cut adjacent to the optic nerve and placed onto Superfrost Plus slides. The slides were blocked with goat serum, incubated with primary antibody overnight at 4°C, washed in phosphate-buffered saline (PBS) then incubated with secondary antibody. The sections are counterstained with DAPI and viewed using a fluorescent microscope (Zeiss Axiovert 200) and images were captured with a digital camera (Axiocam, Zeiss). Photographic and microscopic settings are kept constant for comparisons between antibody and control staining. Controls included tissue not incubated with a primary antibody but otherwise treated identically. For detection of Dkk3 in cultured cells, the cells were grown on sterilized glass cover-slips and then immunostained as above.

For the developmental series, immunohistochemistry was performed essentially as previously described [37]. Mouse retinas were fixed and embedded as above and twelve μm-thick sections were cut. The slides were blocked with 20 mg/ml BSA and 2% donkey serum or 10% goat serum in PBS, and then incubated overnight with goat anti-Dkk3 antiserum at 4°C. Sections were washed in PBS and incubated with secondary antibodies conjugated to a fluorochrome. The antibody selectivity was confirmed by Western blotting whole retina lysates (Figure 2) and by using HEK293 cells transiently expressing Dkk1, Dkk2 and Dkk3.

To immunostain primary Müller glia, cells grown on glass cover-slips were fixed in 4 % paraformaldehyde, permeabilized with 0.05 % Triton-X100, and stained with antibodies detecting Dkk3 (R&D) and glutamine synthetase (Sigma) and the nuclei were counterstained with DAPI.

### Western blot analysis

Eight adult wild-type retinas were pooled to prepare retinal lysates for Western blotting. Retinas were homogenized in RIPA buffer containing proteinase inhibitors, centrifuged at maximum speed in a bench top centrifuge and separated on a 12 % SDS-PAGE gel. To identify secreted Dkk3 in media, MIO-M1 cells were grown to 80% confluency, washed with PBS then incubated with serum-free media overnight. The collected media was acetone precipitated, centrifuged and resuspended in RIPA buffer with proteinase inhibitors and then loaded onto an SDS-PAGE gel. Dkk3 was detected using rat anti-Dkk3 monoclonal antibody (R&D), using chemiluminescence for detection (Amersham Corp., Arlington Heights, IL).

### Statistical Analysis

Values are reported in mean plus standard deviation. Unpaired-*t*-test or one-way analysis of variance and Tukey post-test were used for statistical analyses.

## Authors' contributions

REIN performed the Wnt activation assays and the caspase activity assays. DDH performed the immunohistochemistry of the developing mouse retina. HY assisted with the immunohistochemistry of the adult retina and performed the mouse Müller glia culture experiments. ASH initiated the study, performed HEK293 viability assays and drafted the manuscript. ASH and WJB carried out the research design and project over-sight.

## References

[B1] Moon RT, Kohn AD, De Ferrari GV, Kaykas A (2004). WNT and beta-catenin signalling: diseases and therapies. Nat Rev Genet.

[B2] Hackam AS (2005). The Wnt signaling pathway in retinal degenerations. IUBMB Life.

[B3] Clevers H (2006). Wnt/beta-catenin signaling in development and disease. Cell.

[B4] Chien AJ, Moon RT (2007). WNTS and WNT receptors as therapeutic tools and targets in human disease processes. Front Biosci.

[B5] Logan CY, Nusse R (2004). The Wnt signaling pathway in development and disease. Annu Rev Cell Dev Biol.

[B6] Glinka A, Wu W, Delius H, Monaghan AP, Blumenstock C, Niehrs C (1998). Dickkopf-1 is a member of a new family of secreted proteins and functions in head induction. Nature.

[B7] Zorn AM (2001). Wnt signalling: antagonistic. Curr Biol.

[B8] Krupnik VE, Sharp JD, Jiang C, Robison K, Chickering TW, Amaravadi L, Brown DE, Guyot D, Mays G, Leiby K, Chang B, Duong T, Goodearl AD, Gearing DP, Sokol SY, McCarthy SA (1999). Functional and structural diversity of the human Dickkopf gene family. Gene.

[B9] Mao B, Wu W, Davidson G, Marhold J, Li M, Mechler BM, Delius H, Hoppe D, Stannek P, Walter C, Glinka A, Niehrs C (2002). Kremen proteins are Dickkopf receptors that regulate Wnt/beta-catenin signalling. Nature.

[B10] Semenov MV, Tamai K, Brott BK, Kuhl M, Sokol S, He X (2001). Head inducer Dickkopf-1 is a ligand for Wnt coreceptor LRP6. Curr Biol.

[B11] Brott BK, Sokol SY (2002). Regulation of Wnt/LRP signaling by distinct domains of Dickkopf proteins. Mol Cell Biol.

[B12] Wu W, Glinka A, Delius H, Niehrs C (2000). Mutual antagonism between dickkopf1 and dickkopf2 regulates Wnt/beta-catenin signalling. Curr Biol.

[B13] Li L, Mao J, Sun L, Liu W, Wu D (2002). Second cysteine-rich domain of Dickkopf-2 activates canonical Wnt signaling pathway via LRP-6 independently of dishevelled. J Biol Chem.

[B14] Mao B, Niehrs C (2003). Kremen2 modulates Dickkopf2 activity during Wnt/LRP6 signaling. Gene.

[B15] Caricasole A, Ferraro T, Iacovelli L, Barletta E, Caruso A, Melchiorri D, Terstappen GC, Nicoletti F (2003). Functional characterization of WNT7A signaling in PC12 cells: interaction with A FZD5 × LRP6 receptor complex and modulation by Dickkopf proteins. J Biol Chem.

[B16] Hoang BH, Kubo T, Healey JH, Yang R, Nathan SS, Kolb EA, Mazza B, Meyers PA, Gorlick R (2004). Dickkopf 3 inhibits invasion and motility of Saos-2 osteosarcoma cells by modulating the Wnt-beta-catenin pathway. Cancer Res.

[B17] Kawano Y, Kitaoka M, Hamada Y, Walker MM, Waxman J, Kypta RM (2006). Regulation of prostate cell growth and morphogenesis by Dickkopf-3. Oncogene.

[B18] Monaghan AP, Kioschis P, Wu W, Zuniga A, Bock D, Poustka A, Delius H, Niehrs C (1999). Dickkopf genes are co-ordinately expressed in mesodermal lineages. Mech Dev.

[B19] Barrantes Idel B, Montero-Pedrazuela A, Guadano-Ferraz A, Obregon MJ, Martinez de Mena R, Gailus-Durner V, Fuchs H, Franz TJ, Kalaydjiev S, Klempt M, Holter S, Rathkolb B, Reinhard C, Morreale de Escobar G, Bernal J, Busch DH, Wurst W, Wolf E, Schulz H, Shtrom S, Greiner E, Hrabe de Angelis M, Westphal H, Niehrs C (2006). Generation and characterization of dickkopf3 mutant mice. Mol Cell Biol.

[B20] Harada T, Harada C, Nakayama N, Okuyama S, Yoshida K, Kohsaka S, Matsuda H, Wada K (2000). Modification of glial-neuronal cell interactions prevents photoreceptor apoptosis during light-induced retinal degeneration. Neuron.

[B21] Harada T, Harada C, Kohsaka S, Wada E, Yoshida K, Ohno S, Mamada H, Tanaka K, Parada LF, Wada K (2002). Microglia-Muller glia cell interactions control neurotrophic factor production during light-induced retinal degeneration. J Neurosci.

[B22] Wenzel A, Grimm C, Samardzija M, Reme CE (2005). Molecular mechanisms of light-induced photoreceptor apoptosis and neuroprotection for retinal degeneration. Prog Retin Eye Res.

[B23] Wahlin KJ, Campochiaro PA, Zack DJ, Adler R (2000). Neurotrophic factors cause activation of intracellular signaling pathways in Muller cells and other cells of the inner retina, but not photoreceptors. Invest Ophthalmol Vis Sci.

[B24] Hackam AS, Strom R, Liu D, Qian J, Wang C, Otteson D, Gunatilaka T, Farkas RH, Chowers I, Kageyama M, Leveillard T, Sahel JA, Campochiaro PA, Parmigiani G, Zack DJ (2004). Identification of gene expression changes associated with the progression of retinal degeneration in the rd1 mouse. Invest Ophthalmol Vis Sci.

[B25] Yi H, Nakamura R, Mohamed O, Dufort D, Hackam AS (2007). Characterization of Wnt signaling during photoreceptor degeneration. Invest Ophthal Vis Sci.

[B26] Limb GA, Salt TE, Munro PM, Moss SE, Khaw PT (2002). In vitro characterization of a spontaneously immortalized human Muller cell line (MIO-M1). Invest Ophthalmol Vis Sci.

[B27] Smit L, Baas A, Kuipers J, Korswagen H, van de Wetering M, Clevers H (2004). Wnt activates the Tak1/Nemo-like kinase pathway. J Biol Chem.

[B28] Mikels AJ, Nusse R (2006). Purified Wnt5a protein activates or inhibits beta-catenin-TCF signaling depending on receptor context. PLoS Biol.

[B29] Brault V, Moore R, Kutsch S, Ishibashi M, Rowitch DH, McMahon AP, Sommer L, Boussadia O, Kemler R (2001). Inactivation of the beta-catenin gene by Wnt1-Cre-mediated deletion results in dramatic brain malformation and failure of craniofacial development. Development.

[B30] Mirkovic I, Charish K, Gorski SM, McKnight K, Verheyen EM (2002). Drosophila nemo is an essential gene involved in the regulation of programmed cell death. Mech Dev.

[B31] Hsieh SY, Hsieh PS, Chiu CT, Chen WY (2004). Dickkopf-3/REIC functions as a suppressor gene of tumor growth. Oncogene.

[B32] Chaum E (2003). Retinal neuroprotection by growth factors: a mechanistic perspective. J Cell Biochem.

[B33] Liu C, Peng M, Laties AM, Wen R (1998). Preconditioning with bright light evokes a protective response against light damage in the rat retina. J Neurosci.

[B34] Bush RA, Williams TP (1991). The effect of unilateral optic nerve section on retinal light damage in rats. Exp Eye Res.

[B35] Carmichael J, DeGraff WG, Gazdar AF, Minna JD, Mitchell JB (1987). Evaluation of a tetrazolium-based semiautomated colorimetric assay: assessment of chemosensitivity testing. Cancer Res.

[B36] Hauck SM, Suppmann S, Ueffing M (2003). Proteomic profiling of primary retinal Muller glia cells reveals a shift in expression patterns upon adaptation to in vitro conditions. Glia.

[B37] Libby RT, Hunter DD, Brunken WJ (1996). Developmental expression of laminin beta 2 in rat retina. Further support for a role in rod morphogenesis. Invest Ophthalmol Vis Sci.

